# Quality of Web-Based Sickle Cell Disease Resources for Health Care Transition: Website Content Analysis

**DOI:** 10.2196/48924

**Published:** 2023-12-13

**Authors:** Thomas Annesi, Caren Steinway, Toyosi Oluwole, Steffi Shilly, Dava Szalda, Regina Myers, Jack Chen, Sophia Jan

**Affiliations:** 1 Department of Pediatrics, Donald and Barbara Zucker School of Medicine at Hofstra/Northwell, Hempstead, NY, United States; 2 Division of General Pediatrics, Cohen Children's Medical Center, Northwell Health, New Hyde Park, NY, United States; 3 Columbia University School of Nursing, New York, NY, United States; 4 Division of Hematology, Children’s Hospital of Philadelphia, Philadelphia, PA, United States

**Keywords:** sickle cell, health care transition, readability, Flesch Reading Ease, health care information, adulthood, sickle cell disease, online resource, quality, adolescent, transition, health care service, website quality, online information, Ensuring Quality Information for Patients, EQIP, FRE

## Abstract

**Background:**

Adolescents and young adults with sickle cell disease (SCD) transitioning from pediatric to adult health care face a high-risk period associated with increased use of acute health care services and mortality. Although 59% of American citizens report using the internet for health care information, the quality of web-based, patient-facing resources regarding transition in SCD care has not been evaluated.

**Objective:**

This study aimed to evaluate the quality and readability of web-based health information on SCD, especially as it pertains to the transition to adulthood for inidividuals with SCD. The study also compared the readability and content scores of websites identified in 2018 to those from 2021 to assess any change in quality over time.

**Methods:**

Keywords representing phrases adolescents may use while searching for information on the internet regarding transition in SCD care, including “hydroxyurea” and “SCD transition,” were identified. A web-based search using the keywords was conducted in July 2021 using Google, Yahoo, and Bing. The top 20 links from each search were collected. Duplicate websites, academic journals, and websites not related to SCD health care transition were excluded. Websites were categorized based on the source: health department, hospital or private clinician, professional society, and other websites. Websites were assessed using Health On the Net Foundation code of conduct (HONcode), Flesch Reading Ease (FRE), Flesch-Kincaid Grade Level (FGL), Ensuring Quality Information for Patients (EQIP), and a novel SCD content checklist (SCDCC). EQIP and SCDCC scores range from 0- to 100. Each website was reviewed by 2 research assistants and assessed for interrater reliability. Descriptive statistics were calculated.

**Results:**

Of the 900 websites collected, 67 (7.4%) met the inclusion criteria: 13 health department, 7 hospital or private clinician, 33 professional society, and 14 other websites. A total of 15 (22%) out of 67 websites had HONcode certification. Websites with HONcode certification had higher FRE and EQIP scores and lower FGL scores than those without HONcode certification, reflecting greater readability. Websites without HONcode certification had higher SCDCC scores, reflecting greater clinical content. Only 7 (10%) websites met the National Institutes of Health recommendation of a seventh-grade or lower reading level. Based on EQIP scores, 6 (9%) websites were of high quality. The mean SCDCC score was 20.60 (SD 22.14) out of 100. The interrater reliability for EQIP and SCDCC ratings was good (intraclass correlation: 0.718 and 0.897, respectively). No source of website scored significantly higher mean EQIP, FRE, FGL, or SCDCC scores than the others (all *P*<.05).

**Conclusions:**

Although seeking health care information on the web is very common, the overall quality of information about transition in SCD care on the internet is poor. Changes to current web-based health care information regarding SCD care transitions would benefit transitioning youth by providing expectations, knowledge, skills, and tools to increase self-efficacy.

## Introduction

Sickle cell disease (SCD) is a life-threatening and chronic condition characterized by vaso-occlusion, anemia, and hemolysis [1]. The transition from pediatric to adult health care for young adults with SCD is an especially high-risk period [2-4]. One study found a 2-fold increase in the risk of mortality for patients with SCD aged 19-21 years compared to teenagers aged 16-18 years and a nearly 3-fold higher risk of mortality for young adults aged 22-24 years [3]. Another study that reviewed surveys taken by patients with SCD in pediatric clinics found that a barrier to health care for patients in this population was access to a knowledgeable provider [5]. The first 2 years following transition to adult care in the population with SCD are associated with increased health care use and death [4], theorized to be partly due to poor patient knowledge and skills [6]. Thus, providing youth with SCD with adequate skills and knowledge to manage their own health care is important.

Acquiring the knowledge and skills needed for successfully transitioning from pediatric to adult care has historically fallen to patients and families. Today, 72% of internet users have accessed the internet at least once for health information on the web. Additionally, 92% of American youth access the internet daily [7,8]. As of 2017, an estimated 80% of adolescents and young adults (age 16-24 y) have access to the internet via a computer or smartphone [9]. Additionally, a person living with a chronic disease is more likely to access the internet for health information than the general population of e-patients [10]—patients who use the internet for information related to their condition [11]. Accessibility to web-based health information is important, as perceived website information quality is associated with increased trust for users to choose web-based resources as their main source of health information [12].

Despite patients’ perception that websites are useful for providing health information, the quality of web-based health information that young adults and adolescents with SCD have access to learn about their chronic condition and transition is unknown. There is a vast amount of freely available health information on the internet regarding chronic conditions such as SCD and general transition from pediatric to adult health care, and there are an increasing number of youth accessing it. However, there has not been a content analysis of web-based health information regarding transition to adulthood for patients with SCD. We defined adolescents and young adults as those aged 16-24 years per prior studies investigating the transition period [6,13]. The aims of this study were (1) to evaluate the readability, quality, and content of patient-facing information on the transition to adulthood for patients with SCD available on the internet; (2) to investigate the impact of website source and internet quality certifications on readability, quality measures, and content; and (3) to assess whether readability, quality, and content have improved over the course of 3 years. We hypothesized that the readability, quality, and content of patient-facing information on the transition to adulthood for patients with SCD available on the internet is above the recommended reading level of health information and is of poor quality. Additionally, we did not expect a change in the readability or quality of SCD websites between 2018 and 2021.

## Methods

### Website Search and Inclusion

To collect website data, search terms were established by a team of patients with SCD, pediatricians, internists, social workers, and research staff. Initial search terms were compiled by a group of physicians with expertise in caring for patients transitioning from pediatric to adult health systems. Search terms were distributed to SCD clinical teams across 2 distinct health systems for review and feedback. Final searched terms were as follows: “sickle cell,” “sickle cell disease,” “sickle cell anemia,” “sickle cell transition,” “sickle cell healthcare transition,” “transitional care sickle cell,” “sickle cell transition readiness,” “sickle cell disease medical transition,” “sickle cell anemia resources,” “hydroxyurea,” “hydroxyurea for sickle cell disease,” “sickle cell pediatric to adult care transition,” “sickle cell disease medical resources,” “sickle cell disease symptoms,” and “sickle cell disease treatment.” The Keywords Everywhere extension (Axeman Tech Pvt Ltd) [14] was used to validate that these search terms have been used by other Google users. Keywords Everywhere is a program that provides data on the number of times a search term has been searched per month over the past 15 years within the United States. The program is only available on Google Chrome and Firefox web browsers. Because 65% or more of internet searches take place on Google [15], we assumed that web searches validated on Google will be valid on another platform. Websites were collected in July 2021 using the 3 most used search engines in the United States: Google, Yahoo, and Bing. These search engines make up over 95% of internet searches in the United States [15].

Web searches were performed in an incognito tab to prevent cookies from previous searches from influencing the results. An ad blocker was used to prevent ads from appearing in searches. The first 20 websites per search term were collected. Previous studies have shown that internet users tend to not view search results after the second page of a search [16]. The first 2 pages on all 3 search engines, without ads, include 20 websites. Websites were excluded from the study if they were repeated URLs from a prior search, if their focus was not SCD, if the website contained only videos or links to other pages, or if the website is not accessible to the general public such as an academic publication. Additionally, websites were categorized based on their source: hospital or private clinician, professional society, health department, and other websites. The “other” category included databases such as Wikipedia and WebMD. Lastly, each website was assessed for readability, quality, and transition in SCD care–related content.

To investigate the change in the scores regarding the quality of SCD-related websites over the course of 3 years, unpublished data (S Shilly et al) collected in 2018 were included in this study for comparison. The search terms and inclusion and exclusion criteria were the same in 2018 and 2021. In 2018, SCD health care transition websites were collected from the 5 most used search engines at the time (Google, Yahoo, Bing, DuckDuckGo, and Ask.com), and the data were collected between December 2017 and January 2018.

### Website Readability

Website readability was measured using the Flesch Reading Ease (FRE) and Flesch-Kincaid Grade Level (FGL) formulas. The FRE formula uses word length, the number of syllables, sentence length, and other variables to score an article. Higher scores are associated with greater ease of reading [16]. The FRE formula is scored on a scale from 0-100: scores from 0-29 are considered “very confusing,” 30-49 as “difficult,” 50-59 as “fairly difficult,” 60-69 as “standard” (eighth- and ninth-grade level), 70-79 as “fairly easy,” 80-89 as “easy,” and 90-100 as “very easy” (fifth-grade level) readability [17]. The FGL formula assigns a score that correlates with the US education level a person must achieve to be able to read an article. The National Institutes of Health recommends that medical information should be written at no higher than a sixth-grade reading level, which corresponds to an FGL score below 7 [18,19].

Websites were graded using Microsoft Word, with photographs, figures, and links being removed before being analyzed [20].

The FRE and FGL formulas were chosen as these scores have been used in previous literature to assess health care document readability [21-23]. The scores correspond with a grade level that can be compared to National Institutes of Health recommendations.

### Website Quality

Website quality was measured using the Ensuring Quality Information for Patients (EQIP) tool and Health On the Net Foundation code of conduct (HONcode) certification. The EQIP tool is a 20-item validated instrument that has been used in numerous studies to assess health information quality on the web [24]. It can be used to evaluate the content, identification, and structure of each website. The EQIP tool includes a rating scale of 4 options: yes, partly yes, no, and not applicable. EQIP scores range from 0% to 100%, with higher grades indicating better quality [24]. The EQIP tool was applied to the primary website and the links presented to other sources within it. If a link redirected to an external website, it was not assessed to calculate the EQIP score.

EQIP scores are calculated by dividing a website’s total score across the 20 items by 20 and multiplying this number by 100%. Website quality was scored by 2 research assistants (TA and TO). Intraclass correlations (ICCs) were calculated to measure the agreement between EQIP scores. No consensual agreement has been made regarding cutoff values of EQIP scores to determine website quality. Based on previous studies, a high-quality website was defined as having an EQIP score of ≥75% in this study [25,26].

The EQIP tool was chosen to assess website quality as it is a comprehensive assessment of written medical information. It has been used previously in peer-reviewed literature to assess the quality of information presented on the web [25-27].

HONcode certification identifies whether websites provide quality, objective, and transparent medical information [28]. The Health On the Net Foundation is a not-for-profit nongovernmental organization affiliated with the World Health Organization (WHO). HONcode certification is obtained through a voluntary application that the owner of the website must apply for [29]. Each website is examined by a review committee for the 8 HONcode ethical principles, which are authority (provide qualifications for authors), complementarity (provide information to support, not replace), confidentiality (respect the privacy of site users), attribution (cite the sources and dates of medical information), justifiability (provide justification of claims or balanced and objective claims), transparency (ensure accessibility and provide valid contact details), financial disclosure (provide details of funding), and advertising (clearly distinguish advertising from editorial content). If a website complies with all 8 principles, the site will be given a HONcode seal to place on their page. In our study, each website was assessed for the presence or absence of the HONcode certification.

The presence of HONcode certification was determined using the HONcode search engine [30,31].

The FRE, FGL, EQIP, and SCD content checklist (SCDCC) scores were compared between websites with HONcode certification and those without certification.

HONcode certification was chosen as a method of assessment as the 8 ethical principles the certification is based on align with the 3 pillars of medical ethics: beneficence, nonmaleficence, and justice. HONcode certification has been used in previous literature as a quality assurance method [32,33].

### SCD- and Transition-Related Content

SCD- and transition-related content was assessed using a novel 12-item transition in SCD care-specific content tool (SCDCC), which was produced since no validated tool has been published yet (Textbox 1). The tool was generated using the 6 modifiable factors of transition from the Social-Ecological Model of Adolescent and Young Adult Readiness to Transition [34]. Additionally, items were selected based on the conceptual framework of the chronic care model and the *National Heart, Lung, and Blood Institute SCD Treatment Guidelines* [30,35]. The tool was reviewed by a team of hematologists, psychologists, and other professionals who are experienced and knowledgeable in the transition to adulthood for patients with SCD.

Textbox 1. **The 12 categories graded on the sickle cell disease (SCD) content checklist and the description of the criteria used in their assessment.**

1. Development: evidence of developmental maturity necessary for success in the adult system
2. Knowledge: knowledge related to disease history, health or status, and needs and benefits of transition
3. Skills/Efficacy: skills and self-efficacy needed to manage personal health and transition
4. Beliefs/Expectations: beliefs and expectations related to the transition process
5. Goals: provides achievable goals related to the transition process
6. Relationships: describes relationships among patients, parents, pediatric providers, and adult providers
7. Psychosocial functioning: describes psychological conditions, family functioning, acute crises, stress, and emotions related to the transition process
8. Mood/Pain: describes symptoms of pain crises and emotions related to pain crises
9. Navigating health systems: provides advice on navigating a health system and establishing care during the transition period
10. Self-management: describes increased accountability of obtaining SCD treatment, finding a provider, and managing one’s own health
11. Self-advocacy: describes the need for patient to be involved in their health and aware of their needs
12. Vocational planning: prepares a person with SCD for the workplace

SCDCC scores are calculated by dividing a website’s total score across the 12 items by 12 and multiplying this number by 100%. A total score could range from 0 to 100, with higher scores indicating content more consistent with the above validated frameworks. Each item is scored with either a 0 for item not present, 1 for item clearly present, or 0.5 for item not clearly present but alluded to. Website content was scored by 2 research assistants (TA and TO).

The purpose of adding the SCDCC as an analytic tool was to supplement the EQIP score as a method to better assess written SCD material.

### Statistical Analysis

The FRE, FGL, EQIP, and SCDCC scores were compared between websites with HONcode certification and those without certification.

Websites were categorized based on their source. Websites ending in “.gov” were designated as health department websites. Websites associated with a hospital or system were designated hospital or private clinician websites. Websites were categorized as professional society websites if they were produced by a medically oriented professional association. Websites designated as “other” did not fit into the aforementioned categories. FRE and FGL scores were calculated by copying and pasting the text on each website into a word-processing program. An extension in the word-processing program that calculates readability was used. EQIP scores were measured using a 20-point scale established by Moult et al [24]. The score out of 20 items was then divided by 20 and multiplied by 100% to get a percentage of EQIP items that the website possessed. Similarly, SCDCC scores were calculated on a 12-point scale, divided by 12 and multiplied by 100%. Google sheets with the *XLMiner ToolPak* (Frontline Systems Inc) was used to assess ANOVA and 2-tailed *t* tests. ANOVA was used to measure statistically significant differences in mean FRE, FGL, EQIP, and SCDCC scores between each website source. *t* tests were used to measure statistically significant differences between the mean scores of websites with and without HONcode certification. *t* tests were also used to measure statistically significant differences in mean FRE, EQIP, and SCDCC scores for websites in 2018 and 2021.

## Results

### Website Search

In 2021, a total of 900 websites were collected, with 67 (7.4%) meeting the inclusion criteria: 13 health department, 7 hospital or private clinician, 33 professional society, and 14 other websites.

### FRE and FGL Evaluation

The mean FRE score among all websites was 54.64 (SD 10.48; range 24.4-78.6), indicating that the websites were difficult to read (the recommended range is >70). There were no significant differences in FRE scores between website sources (*F*_3_=0.262; *P*=.85; Table 1). The reading difficulty of each website was stratified based on their FRE score. Of the 67 websites, only 7 (10%) were “fairly easy to read” (FRE score 70-79). Of those 7 websites, 1 was a hospital or private clinician website, 2 were health department websites, 2 were professional society websites, and 2 were other websites.

**Table 1.** Readability scores.

Score	Website source				
	All sources	Hospital or private clinician	Health department	Professional society	Other
FRE^a^, mean (SD)	54.64 (10.48)	54.56 (10.65)	55.55 (9.54)	55.67 (9.47)	56.03 (18.67)
FGL^b^, mean (SD)	9.72 (1.96)	9.84 (2.83)	9.54 (1.94)	9.57 (2.17)	8.99 (3.87)

a FRE: Flesch Reading Ease.

b FGL: Flesch-Kincaid Grade Level.

The mean FGL score among all websites was 9.72 (SD 1.96; range 1.94-19.2), also indicating that the websites were above the recommended seventh-grade reading level. There were no significant differences in FGL scores between website sources (*F*_3_=0.341; *P*=.69; Table 1). Of the 67 websites, only 8 (12%) had an FGL score in the recommended range of <7. Of those 8 websites, 4 were hospital or private clinician websites, 1 was a health department website, 1 was a professional society website, and 2 were other websites.

### EQIP Evaluation

The mean EQIP score was 47.18 (SD 13.00) for all websites. The mean EQIP scores for each website source were as follows: hospital or private clinician, 49.30 (SD 12.32); health department, 52.88 (SD 13.89); professional society, 51.95 (SD 12.45); and other, 59.82 (SD 8.82). There were no significant differences in EQIP scores between website sources (*F*_3_=1.96; *P*=.12; Table 2). Of the 67 websites, only 1 (1%) achieved an EQIP score over 75. The interrater reliability of EQIP scores calculated using ICC was 0.718, which is considered acceptable for interrater reliability.

**Table 2.** Quality scores.

Score	Website source				
	All sources	Hospital or private clinician	Health department	Professional society	Other
EQIP^a^, mean (SD)	47.18 (13.00)	49.30 (12.32)	52.88 (13.89)	51.95 (12.45)	59.82 (8.82)
SCDCC^b^, mean (SD)	20.60 (22.14)	37.32 (28.48)	26.12 (19.88)	22.79 (17.60)	19.05 (6.74)

a EQIP: Ensuring Quality Information for Patients.

b SCDCC: sickle cell disease content checklist.

The EQIP items most frequently graded as “yes” or “partly yes” were “Is the tone respectful” and “Is the information presented in logical order.” The EQIP items least frequently graded as “yes” or “partly yes” were “Does the document have a named space for the reader to make notes” and “Does the document say whether patients and/or family members were involved or consulted in its production.”

### SCDCC Evaluation

The mean website content score for SCD websites was 20.60 (SD 22.14). The mean SCDCC scores for each website source were as follows: hospital or private clinician, 37.32 (SD 28.48); health department, 26.12 (SD 19.88); professional society, 22.79 (SD 17.60); and other, 19.05 (SD 6.74). The interrater reliability of SCDCC scores calculated using ICC was 0.897. There were no significant differences in SCDCC scores between website sources (*F*_3_=2.32; *P*=.08; Table 2).

The SCDCC items most frequently graded as “yes” or “partly yes” were “Knowledge” and “Mood/Pain.” The SCDCC items most frequently graded as “no” were “Vocational Planning” and “Goals.” Less than 25% of websites contained information related to “Skills/Efficacy” (16/67, 24%), “Self-Advocacy” (15/67, 22%), “Relationships” (12/67, 18%), “Vocational Planning” (6/67, 9%), “Development” (15/67, 22%), and “Goals” (7/67, 10%).

### HONcode Certification

Of the 67 websites reviewed, 15 (22%) had a HONcode certification. Of the 15 websites who had the certification, 3 (20%) were developed by a health department, 2 (13%) were developed by a hospital or private clinician, 4 (27%) were developed by a professional society, and 6 (40%) were developed by other sources.

Websites that were HONcode certified had significantly higher FRE (*P*=.02) and lower FGL (*P*=.001) scores (greater readability) and higher EQIP (*P*=.004) scores (higher quality).

There were no significant differences between the SCDCC scores (range of contents) of websites with HONcode certification versus those without HONcode certification (*P*=.30; Table 3).

**Table 3.** Comparison of the mean FRE^a^, FGL^b^, EQIP^c^, and SCDCC^d^ scores for websites with HONcode^e^ certification and websites without HONcode certification.

Score	Websites with HONcode certification, mean (SD)	Websites without HONcode certification, mean (SD)	*P* value
FRE	60.37 (11.81)	53.51 (9.90)	.02
FGL	8.12 (1.98)	10.08 (2.40)	.001
EQIP	58.08 (11.59)	48.31 (12.44)	.004
SCDCC	20.56 (10.38)	28.21 (25.50)	.30

a FRE: Flesch Reading Ease.

b FGL: Flesch-Kincaid Grade Level.

c EQIP: Ensuring Quality Information for Patients.

d SCDCC: sickle cell disease content checklist.

e HONcode: Health On the Net Foundation code of conduct.

### Comparison Between 2018 and 2021 Data

In 2018, a total of 1924 websites were collected, with 92 (4.8%) meeting inclusion criteria: 11 health department, 38 hospital or private clinician, 21 professional society, and 22 other websites. In all, 31 websites identified in 2018 were also identified in 2021. When the quality of data was compared between 2018 and 2021, websites still scored poorly for FRE, EQIP, and SCDCC (Table 4). Overall, the quality of websites did not improve between the 2 data collection periods. Compared to 2018, websites in reviewed 2021 had statistically lower EQIP scores (*P*=.006), indicating a slight degradation of website quality over time. In both 2018 and 2021, websites were fairly difficult to read on average as assessed by the FRE formula. There was also a lack of HONcode-certified websites in both 2018 and 2021. Compared to 2021, more websites had HONcode certification in 2018 (2021: 15/67, 22% vs 2018: 26/92, 28%).

**Table 4.** Comparison of the mean FRE^a^, EQIP^b^, and SCDCC^c^ scores for websites from 2018 to 2021.

Score	Year, mean (SD)		*P* value
	2018	2021
FRE	51.8 (13.6)	55.1 (11.1)	.10
EQIP	56.8 (11.0)	51.6 (12.5)	.006
SCDCC	20.8 (18.1)	30.1 (24.1)	.008

a FRE: Flesch Reading Ease.

b EQIP: Ensuring Quality Information for Patients.

c SCDCC: sickle cell disease content checklist.

Similar to the results from 2021, websites with HONcode certification in 2018 were associated with significantly higher FRE scores (*P*=.02). The mean FRE score for websites with HONcode certification was 56.57 (SD 11.12) and the mean FRE score for websites without HONcode certification was 50.01 (SD 14.21). There were no significant differences between EQIP and SCDCC scores (*P*=.18 and *P*=.44, respectively).

## Discussion

### Principal Findings

This is one of the first studies to conduct a content analysis of web-based health information regarding the transition to adult care for patients with SCD. Previous studies have shown that the quality of health information available on the internet is variable and unregulated [31]. Our study is consistent with this finding. Our study indicated that the overall quality of information on SCD transition to adult care on the internet does not meet the recommended seventh-grade or lower reading level, which is consistent with other studies on SCD websites [36]. On average, websites containing information on the transition in SCD care were fairly difficult to read. These websites also failed to score highly on the EQIP and SCDCC assessments. Website analysis revealed few HONcode certifications, which is consistent with other studies on health information [32,33]; few websites that met the recommended reading level; and few websites of high quality based on EQIP and SCDCC scores. Websites hosted by academic centers, hospitals, or governmental sources were not comparatively better in readability, quality, or content. For the small proportion of websites that obtained HONcode certification, certification is associated with improved readability and quality, without sacrifices in content, although the content based on the SCDCC score was relatively poor overall. HONcode certification may be a helpful check for website readability and quality, given the association of HONcode certification on the measures in our study, and HONcode certification can be obtained for any website by requesting for review over the web.

Furthermore, website quality did not significantly improve between 2018 and 2021. The poor scores for websites in both 2018 and 2021 point to the need for the improvement of web-based health resources for adolescents and young adults with SCD. We acknowledge that EQIP and SCDCC scores increased between 2018 and 2021; however, the mean scores were of poor quality in both years. Academic centers, governmental agencies, and other organizations should ensure their websites are up to date and comprehensive to increase the readability and quality of content for patients with SCD. The web-based sources that were analyzed in this study lack a well-rounded view of a patient’s experience with the SCD health care transition process. Although these websites provide factual information, they are not written to the level of an average reader nor do they provide clear expectations and a holistic view of a patient with SCD. The consequences of poorly designed websites can include decreased patients’ perception of usefulness and trust in web-based information and withered patient-physician relationships [12,30,35]. According to previous studies, the quality of health websites remains poor and inconsistent, and web-based information is being used more among patients as it can improve the patient-physician relationship [12,36-39]. It is important for there to be improvements in the quality of the websites that would allow youth with SCD to learn to manage their own health.

The readability of websites can be improved by using shorter sentences and avoiding medical jargon. When medical terms need to be used, they should be explained in terms that can be understood by the average person. The quality of websites can be improved by writers using the EQIP tool as a guideline. Additionally, we believe the SCDCC can act as a guideline for writers to provide well-rounded information regarding the process of transitioning their SCD care. Involving people with SCD in the writing process of these websites can also help to improve the quality of websites [40], as their feedback would be invaluable.

Health information for transitioning patients with SCD needs to be tailored to adolescent and young adult patients by aligning with their cognitive skills and providing well-rounded information [41]. Gaps in adolescent and young adult patients’ knowledge pose a threat to their health during the vulnerable transition time period. It has been established that this is a time of increased morbidity and mortality [2-4] and that knowledge about SCD is a barrier to care [5]. We identified that web-based, patient-facing information on the transition of SCD care is, on average, fairly difficult to read and generally of poor quality. Physicians should be knowledgeable that their patients with SCD will likely use the internet to obtain medical information and that there are gaps in web-based information. Reliable, quality websites should be given to patients for reference, and a discussion should be initiated between patients with SCD and their providers to identify the knowledge the patient has and bridge any gaps in their knowledge.

### Limitations

Our study only included websites that presented information in English. Future studies should be conducted to evaluate websites in other languages that appear in search results. Additionally, using incognito mode to search for websites does not prevent the location from affecting search results. The websites that were collected may have been influenced by our location in New York state. Websites from other countries and states may have been excluded. A virtual private network can be used in future studies to access websites from other areas of the world. Our study is time sensitive to a single point in time. Websites are updated or changed every 27 months on average, and HONcode certification and FRE, FGL, EQIP, and SCDCC scores are subject to change [42]. HONcode certification is a voluntary process by the site owner, meaning that websites may meet the HONcode criteria but have not been certified due to not applying for certification [29]. Additionally, there is no resource available that lists sites that have applied for HONcode certification but did not meet the criteria. Due to the subjective nature of EQIP and SCDCC scoring, the scores are subject to variation, although no significant variations were seen between the 2018 and 2021 websites. The SCDCC has not been validated as a measure of the quality of SCD-specific information. We believe that the scale offers a valuable insight into SCD-specific, web-based information that the EQIP tool cannot offer. This study investigated the quality of SCD websites and did not investigate other mediums of health information available on the internet such as social media. Future studies investigating the quality of information presented on platforms other than websites would provide valuable information given the popularity of social media. Despite the inherent limitations present in this study, we believe that the observations of the study are significant and show that the quality of web-based information regarding preparation for the transition of SCD care is not satisfactory.

### Conclusions

Many websites available for patients with SCD transitioning from pediatric to adult care lack a well-rounded view of the experiences and health care needs of these patients. Additionally, these websites can be fairly difficult to read. Websites can be improved by using shorter sentences, limiting the length of sentences, and providing a more comprehensive view of the SCD transition of care process. With these changes, improvements in self-management of adolescents and young adults with SCD transitioning from pediatric to adult care may be seen.

## Abbreviations

EQIP: Ensuring Quality Information for Patients
FGL: Flesch-Kincaid Grade Level
FRE: Flesch Reading Ease
HONcode: Health On the Net Foundation code of conduct
ICC: intraclass correlation
SCD: sickle cell disease
SCDCC: sickle cell disease content checklist
WHO: World Health Organization
